# Luteolin attenuates Wnt signaling via upregulation of FZD6 to suppress prostate cancer stemness revealed by comparative proteomics

**DOI:** 10.1038/s41598-018-26761-2

**Published:** 2018-06-04

**Authors:** Kun Han, Tingyuan Lang, Zhiqi Zhang, Yi Zhang, Yongning Sun, Zan Shen, Roger W. Beuerman, Lei Zhou, Daliu Min

**Affiliations:** 1 Department of Medical Oncology, The Affiliated 6th People’s Hospital of Shanghai Jiaotong University, Shanghai, 200233 China; 2 Key Laboratory of Clinical Laboratory Diagnostics (Ministry of Education), College of Laboratory Medicine, Chongqing Medical University, Chongqing, 400016 China; 3 Singapore Eye Research Institute, The Academia, 20 College Road, Discovery Tower level 6, Singapore, 169856 Singapore; 4 Department of Ophthalmology, Yong Loo Lin School of Medicine, National University of Singapore, 1E Kent Ridge Road, NUHS Tower Block Level 7, Singapore, 119228 Singapore; 5 Neuroscience and Behavioral Disorders Program, Duke-NUS Graduate Medical School, 8 college Road, Singapore, 169857 Singapore

## Abstract

The mechanisms underlying luteolin-induced inhibition of prostate cancer (PCa) stemness have remained elusive. Here, we report that luteolin suppresses PCa stemness through Wnt signaling by upregulation of FZD6 (frizzled class receptor 6). Luteolin inhibits PCa cell proliferation, migration, self-renewal as well as the expression of prostate cancer stem cell markers *in vitro*. Through iTRAQ-based quantitative proteomics study, we identified 208 differentially expressed proteins in luteolin-treated PC-3 cells. Subsequent mechanistic analysis revealed that luteolin inhibits Wnt signaling by transcriptional upregulation of FZD6, and thereby suppressing the stemness of PCa cells. Furthermore, we identified FZD6 as a tumor suppressor that can abolish PCa stemness. In summary, our findings demonstrate that suppression of Wnt signaling by upregulation of FZD6 is a mechanism underlying luteolin-induced inhibition of PCa stemness. Our work suggests a new therapeutic strategy against human prostate cancer caused by aberrant activation of Wnt signaling.

## Introduction

Prostate cancer (PCa) is the second leading cause of cancer death among men in the western countries^1^. So far, there is no real cure beyond surgery and radiation. Early stages of prostate cancer can be controlled with hormone ablation therapy^2^. However, over time when prostate cancer overcomes its hormone dependence, it becomes castration-resistant prostate cancer (CRPC) and metastasizes to other organs^3^. Accumulating evidence implied that cancer stem cells (CSCs) play a critical role in CRPC development and metastasis of prostate cancer^4–6^. Inhibition of prostate cancer CSCs, therefore, has emerged as a new therapeutic strategy^7,8^. On the other hand, chemotherapy drugs and other novel drugs used for prostate cancer, such as enzalutamide and abiraterone, are aggressive and all have adverse side effects^9^; Thus, natural compounds, which inhibit CSCs may prove to be promising agents for prostate cancer therapy^10^.

Luteolin (3′,4′,5,7-tetrahydroxyflavone), a common dietary flavonoid, has been shown its *in vitro* and *in vivo* anti-cancer activity against various cancer cells including prostate cancer^11–13^. Evidence from human, animal model and cell-based studies have demonstrated that luteolin inhibits cancer initiation and progression by interfering with transcription factors and kinases, regulating cell cycle, apoptosis and inhibiting cell transformation, migration, invasion, and angiogenesis^14–17^. Importantly, luteolin has been used as Traditional Chinese Medicine for treatment of hypertension and inflammatory diseases^18,19^, indicating its safety for clinical use. Thus, luteolin could be a good candidate for cancer therapy.

Despite numerous studies, the mechanism of luteolin’s anti-prostate cancer activity is still elusive. Proteomics has been widely used for identifying new molecular targets and elucidating the mechanisms of anticancer drugs^20^. By comparing changes in protein expressions upon drug treatment, proteomics provides rich information on understanding mechanism-of-action of a drug and its toxicity^21^.

In order to enhance the understanding of the molecular mechanisms of luteolin treatment, in this study, we investigated the effects of luteolin on the proteomic profile of prostate cancer cells. We showed that a negative regulator of β-catenin transcriptional activity, FZD6 (frizzled class receptor 6), is one of the key regulators related to luteolin treatment; it inhibits Wnt signaling pathway and the stemness of prostate cancer cells. Our findings may aid improvement of translational application of luteolin and development of novel anti-prostate cancer drugs.

## Results

### Luteolin inhibits the stemness of PCa cells *in vitro*

**Figure 1:**
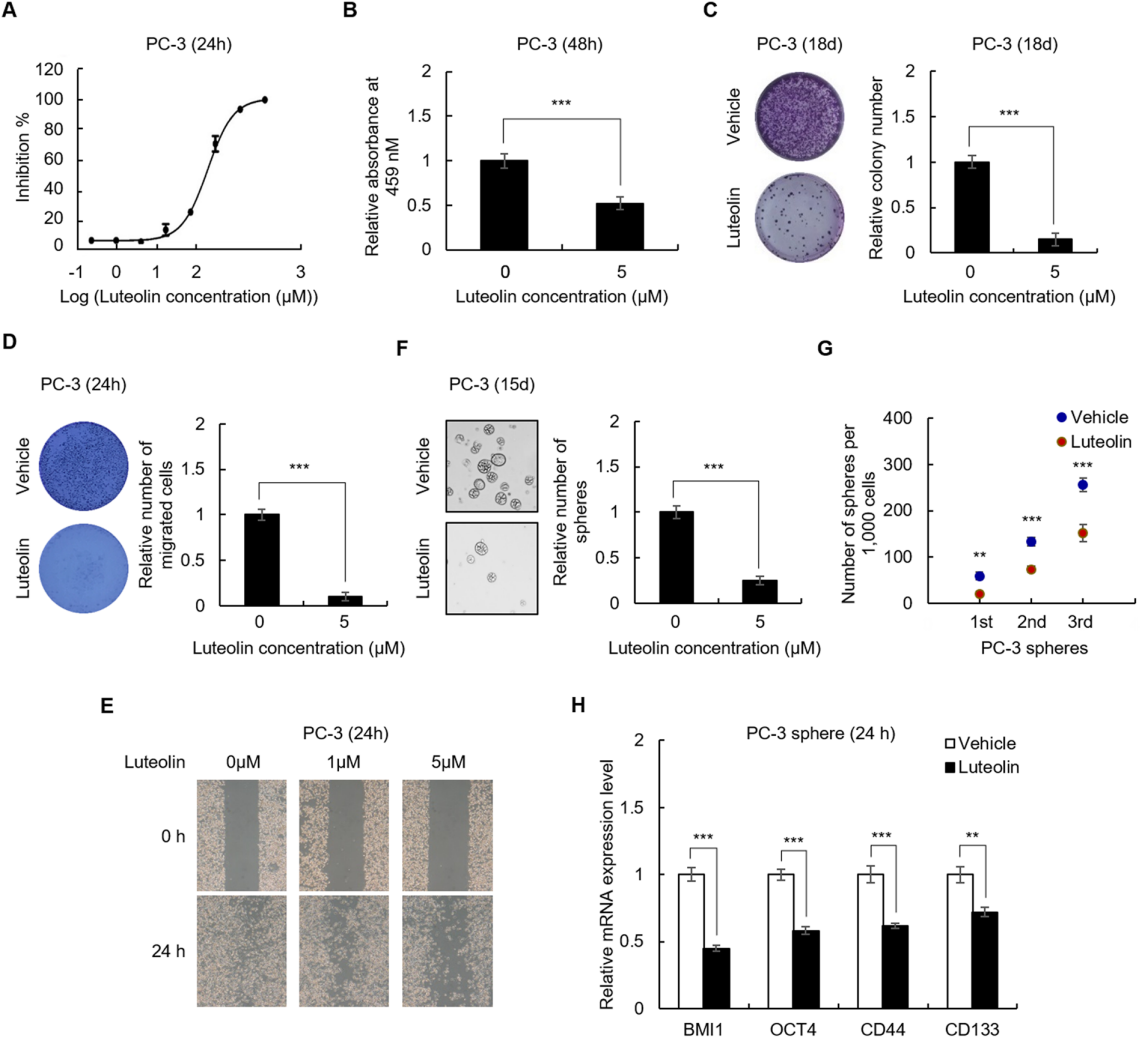
Luteolin inhibits the stemness of PC-3 cells. (**A**) Luteolin dose-response curves of PC-3 cells. PC-3 cells were treated with indicated concentration of luteolin for 24 h, the cell viability was determined by CCK8 assay. IC50 value was obtained by fitting a four-parameter dose–response curve. (**B**) CCK8 assay of PC-3 cells treated with 5 μM of luteolin or equal volume of vehicle for 48 h. (**C**) Colony formation assay of PC-3 cells treated with 5 μM of luteolin or equal volume of vehicle for 18 days. (**D**,**E**) Luteolin inhibits the migration of PC-3 cells. PC-3 cells were treated with indicated concentration of luteolin for 24 h. Cell migration activity was measured by CytoSelect Fluorometric 8 µM Transwell Migration Assay plate (**D**) and wound healing assay (**E**), (**F,G**) Sphere formation assay of PC-3 cells treated with 5 μM of luteolin or equal volume of vehicle for 15 days. The number of 1st, 2nd and 3rd passaged spheres was counted (**G**), (**H**) qRT-PCR of indicated gene in PC-3 spheres treated with 5 μM of luteolin or equal volume of vehicle for 12 h. Data are representative of at least three independent experiments.

Maximal non-toxic chemical concentration that causes an acceptably small effect in the cells is usually chosen for cell-based experiments^22^. We thus first performed limiting dilution analysis (LDA)^23^ to determine the maximal non-toxic concentration of luteolin on PC-3 cells, which is the most commonly used cell line for prostate cancer research. As shown in Fig. 1A, the half maximal inhibitory concentration (IC50) is 25.25 μM and the maximal non-toxic concentration is about 5 μM. Next, we found that 48 h treatment with 5 μM of luteolin significantly inhibited the proliferation of PC-3 cells (Fig. 1B) and 18 days treatment suppressed the clonogenicity of the cells (Fig. 1C), which suggested that the maximal non-toxic dose of luteolin leads to molecular alterations involved in proliferation.

We then investigated whether the maximal non-toxic dose of luteolin inhibits the stemness of PCa cells. We first examined the effect of luteolin on the migration of PC-3 cells. As shown in Fig. 1D,E, treatment with 5 μM of luteolin for 24 h significantly inhibited the migration of PC-3 cells as indicated by transwell and wound healing assay. Sphere formation in suspension culture is a prominent characteristic of cancer stem cells, we thus next determined the effect of luteolin on sphere formation of PC-3 cells^24,25^. As shown in Fig. 1F,G, the treatment obviously suppressed the spherogenicity and reduced self-renewal capacity on serial passage in the cells. Moreover, the expression of PCa stem cell markers (including CD44, CD133, OCT4 and BMI1)^5,6,24,25^ in PC-3 spheres were reduced by luteolin as indicated by qRT-PCR (Fig. 1H). These results were confirmed by primary PCa cells (Supplementary Fig. S1A–C), which suggested that treatment with the maximal non-toxic dose of luteolin suppresses the stemness of PCa cells.

Taken together, above results demonstrated that luteolin inhibits the stemness of PCa cells *in vitro* and treatment with the maximal non-toxic dose of luteolin results in molecular alterations involved in proliferation, migration and stemness in PCa cells, but does not cause cell death, and thereby is appropriate for study of mechanism-of-action of luteolin against PCa.

### Quantitative Proteomic Profiling of PC-3 Cells with and without Luteolin Treatment

**Figure 2:**
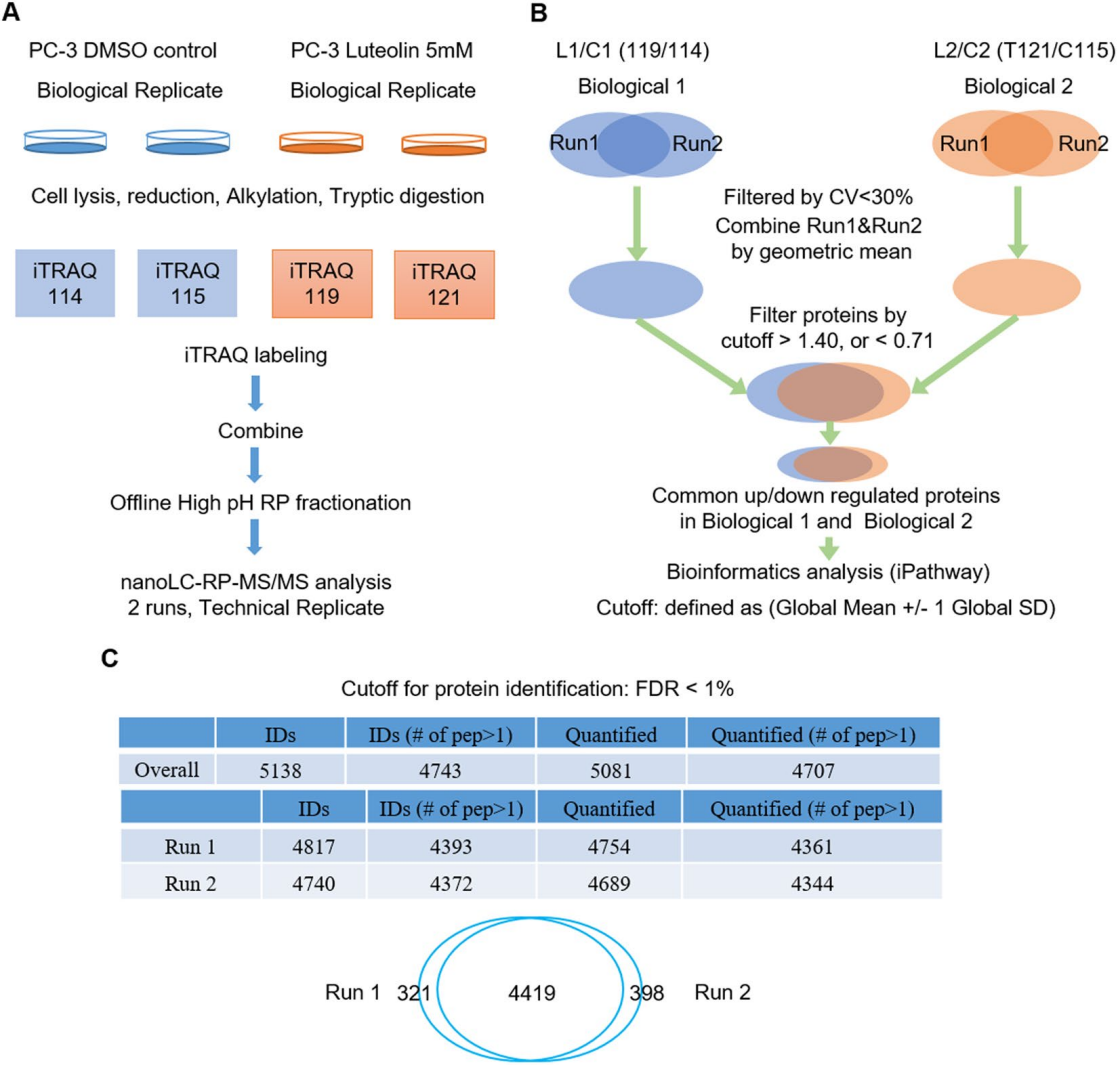
Proteomic analysis of PC-3 cells with and without luteolin treatment. (**A**) Workflow of the experiment. PC-3 cells were treated with and without luteolin. After tryptic digestion and iTRAQ labeling, the peptide mixture was fractionated into 10 fractions using high pH reversed-phase HPLC followed by nanoLC-RP-MS/MS. (**B**) iTRAQ quantitative analysis. Data with coefficient of variation less than 30% between two technical runs were kept for further analysis. Only proteins with fold change of >1.4 or <0.71 and were observed in both biological replicates are considered as differentially expressed proteins. (**C**) Results of proteomic analysis. In total, 5138 unique proteins (4743 proteins identified with at least two peptide fragments) were identified with high confidence (<1% false discovery rate (FDR)). 5081 proteins were quantifiable (4707 proteins were quantifiable with at least two peptide fragments). Highly reproducible results were observed between two technical runs with >86% of proteins (4419 out of 5138 proteins) seen in both runs.

**Figure 3:**
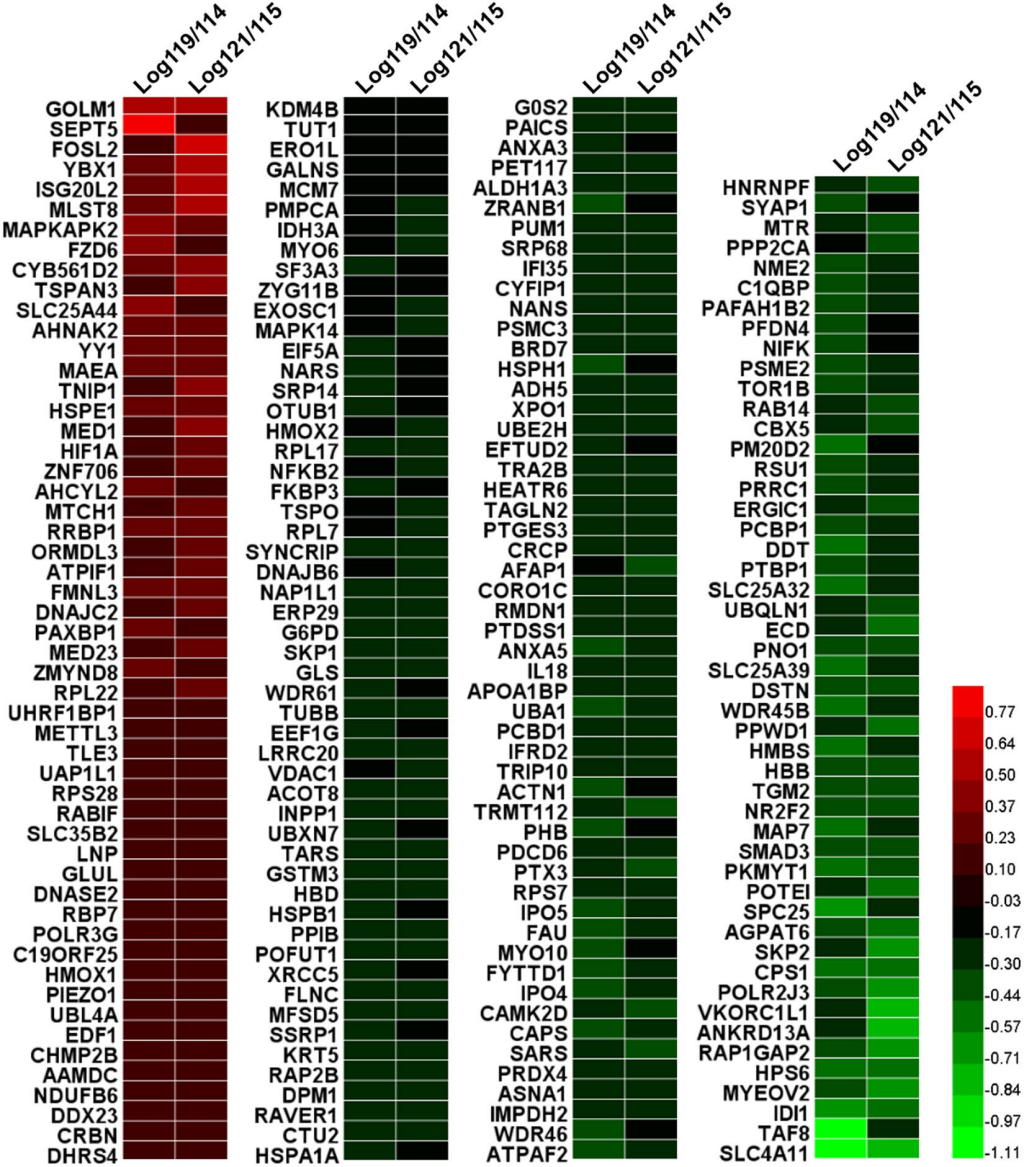
Differentially expressed proteins. Luteolin regulates the expressions of 208 proteins in PC-3 cells. Comparative proteomic analysis were performed using PC-3 cells with and without luteolin treatment by iTRAQ method. Data with coefficient of variation less than 30% between two technical runs were kept for further analysis. Proteins with fold change of >1.4 or <0.71 and were observed in both biological replicates are considered as differentially expressed proteins.

To examine the protein expression profiles that were associated with luteolin treatment, we performed a comparative proteomic analysis. A schematic description of the experimental design and data process strategy is presented in Fig. 2A,B. After tryptic digestion and iTRAQ labeling, the peptide mixture was fractionated into 10 fractions using high pH reversed-phase HPLC. These 10 fractions were further analyzed by nanoLC-RP-MS/MS (each fraction was injected two times). In total, 5138 unique proteins (4743 proteins identified with at least two peptide fragments) were identified with high confidence (<1% false discovery rate (FDR)). Among them, 5081 proteins were quantifiable (4707 proteins were quantifiable with at least two peptide fragments). Highly reproducible results were observed between two technical runs with >86% of proteins (4419 out of 5138 proteins) seen in both runs (Fig. 2C). iTRAQ quantitative analysis was based on the stringent criteria shown in Fig. 2B. The cutoff for up- or down-regulated was defined as Global Mean ± 1 Global SD. Data with a coefficient of variation less than 30% between two technical runs were kept for further analysis. Only proteins with a fold change of >1.4 or <0.71 and were observed in both biological replicates are considered as differentially expressed proteins. A list of 208 differentially expressed proteins (53 up-regulated and 155 down-regulated) were selected for further bioinformatics analysis (Fig. 3).

### Bioinformatic analysis and target identification

**Figure 4:**
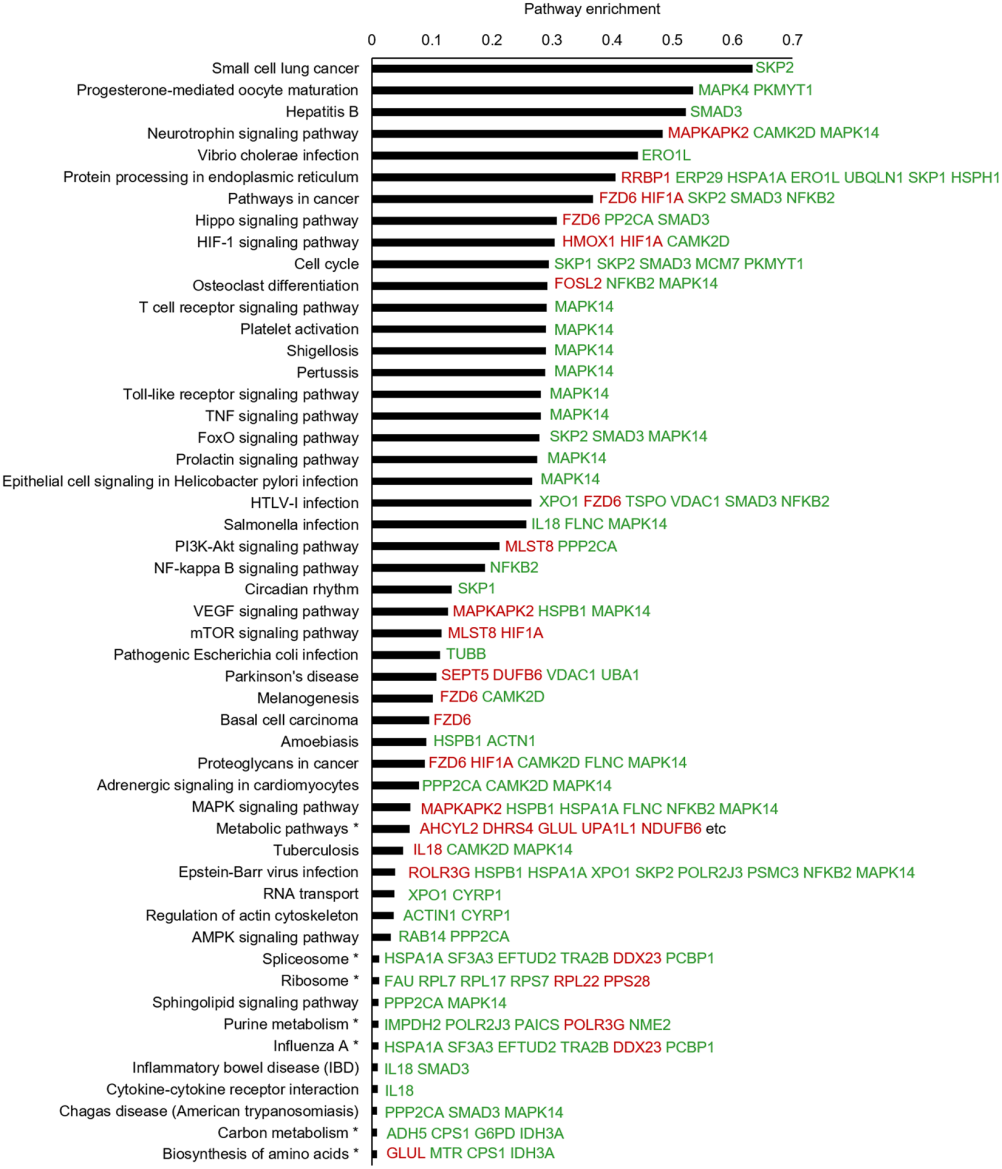
Pathway analysis of the differentially expressed proteins. The gene symbols of 208 differentially expressed proteins were subjected to iPathwayGuide online software for pathway analysis. Top 50 impacted pathways were listed.

The differentially expressed proteins were subsequently subjected to pathway analysis using iPathwayGuide online software^26^ to yield insights into biological functions regulated by luteolin. As shown in Fig. 4, the enriched pathways of differentially expressed proteins include several essential signaling pathways involved in PCa initiation and progression, such as Wnt, Hippo, HIF-1, TNF, FoxO, PI3K/AKT, VEGF, mTOR, and MAPK, suggesting that luteolin regulates these pathways. Gene ontology analysis according to the cluster of the gene ontology terms^27^ was also performed (Supplementary Fig. S2) and the results indicated a significant enrichment of genes related to chromatin organization (Supplementary Table S1), mRNA processing (Supplementary Table S2) as well as translation initiation, elongation and termination (Supplementary Table S3), which suggested that luteolin may regulate the expression of its target genes mainly by regulating transcription and translation.

### FZD6-mediated suppression of Wnt signaling is critical for luteolin inhibiting PCa stemness

**Figure 5:**
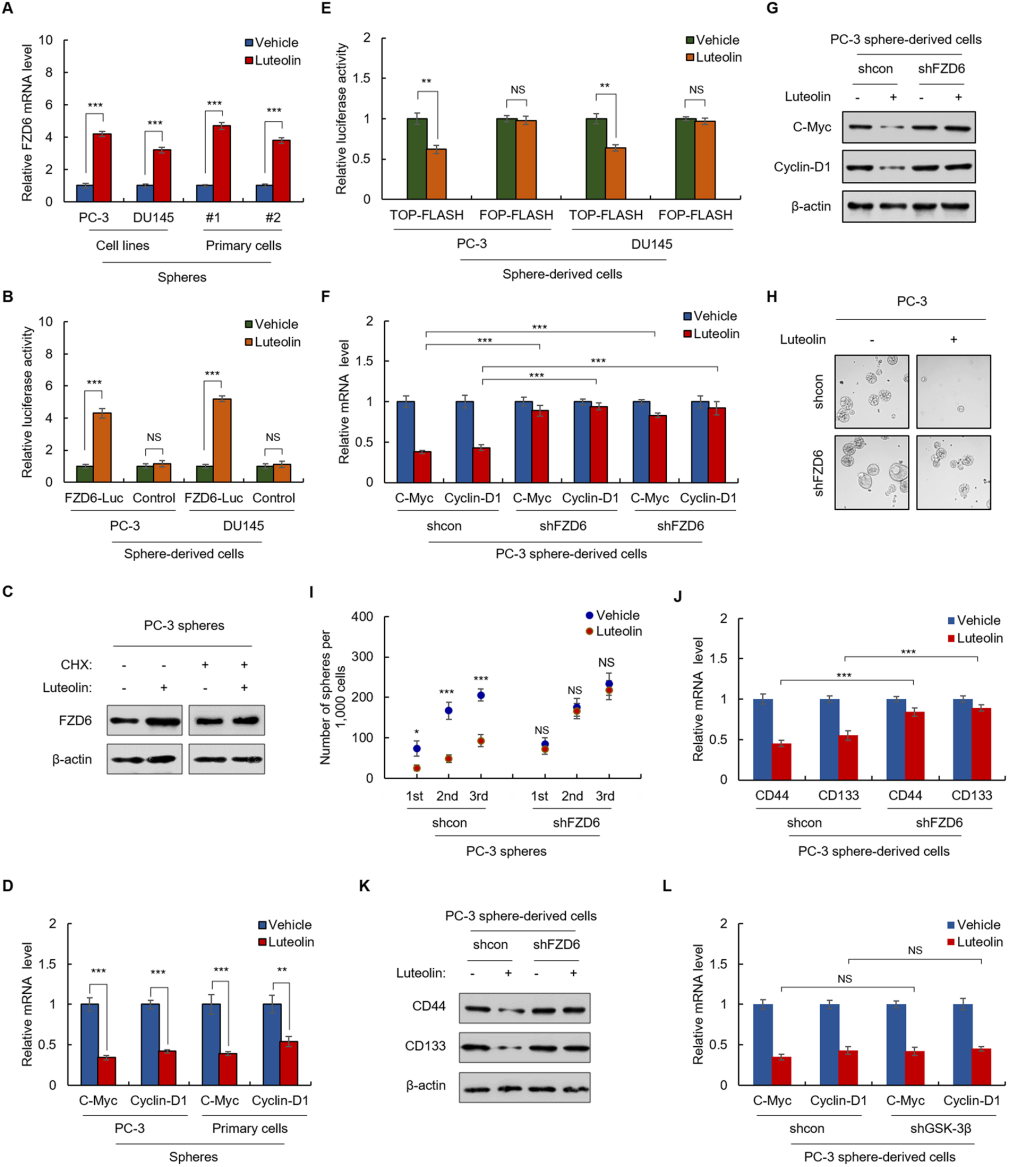
FZD-mediated suppression of Wnt signaling is critical for luteolin inhibiting PCa stemness. (**A–C**) Luteolin upregulates FZD6 at the transcriptional level in PCa cells. (**A**) qRT-PCR of FZD6 in indicated PCa spheres treated with 5 μM of luteolin or equal volume of vehicle for 24 h. (**B**) Indicated cells were transfected with FZD6 promoter-driven luciferase reporter plasmid. Luciferase activities were measured after 24 h treatment with or without 5 μM of luteolin. Relative luciferase activity was normalized by that in cells transfected with the control vector. (**C**) Western blot of FZD6 in cycloheximide-pretreatment PC-3 spheres after 24 h treatment with or without 5 μM luteolin. (**D**,**E**) Luteolin suppresses Wnt signaling in PCa cells. (**D**) qRT-PCR of C-Myc and Cyclin-D1 in PC-3 and primary cells treated with or without 5 μM of luteolin for 24 h. (**E**) PC-3 and DU145 cells were transfected with indicated plasmids. Luciferase activities were measured after 24 h treatment with or without 5 μM of luteolin. Relative luciferase activity was normalized by that in cells transfected with the control vector. (**F**,**G**) Upregulation of FZD6 is necessary for luteolin inhibiting Wnt signaling in PCa cells. qRT-PCR (**F**) and western blot (**G**) of C-Myc and Cyclin-D1 in FZD6-depleted PC-3 sphere-derived cells and control cells treated with or without 5 μM of luteolin for 24 h. (**H–K**) Upregulation of FZD6 is necessary for luteolin inhibiting the stemenss of PCa cells. (**H**,**I**) Sphere formation of FZD6-knockdown PC-3 cells and control cells treated with or without 5 μM of luteolin. (**I**) The number of 1st, 2nd and 3rd passaged spheres was counted. (**J**,**K**) qRT-PCR (**J**) and western blot (**K**) of CD44 and CD133 in luteolin-treated and non-treated FZD6-knockdown PC-3 sphere-derived cells and control cells. (**L**) Luteolin-induced suppression of Wnt signaling in PCa cells is GSK-3β independent. qRT-PCR of C-Myc and Cyclin-D1 in luteolin-treated and non-treated GSK-3β-knockdown PC-3 sphere-derived cells and control cells. Data are representative of at least three independent experiments.

Because of our observed luteolin-induced upregulation of FZD6 (Figs 3 and 4), a negative regulator in Wnt signaling pathway^28^, and the fact that Wnt signaling plays a central role in tumorigenesis and crosstalks with multiple tumorigenic signaling pathways^29^, we next confirmed the regulatory effect of luteolin on FZD6 as well as Wnt signaling in PCa cells and investigated its role in luteolin-induced suppression of PCa stemness. As shown in Fig. 5A, luteolin treatment resulted in a significant increase in the mRNA level of FZD6 in PC-3 spheres as indicated by qRT-PCR. In addition, results from luciferase reporter assay revealed that luteolin increased the transcriptional activity of FZD6 promoter in PC-3 sphere-derived cells (Fig. 5B). These results suggested that luteolin upregulates the expression of FZD6 at the transcriptional level. Next, to investigate the effect of luteolin on the stability of FZD6, we tested the protein level of FZD6 in luteolin-treated and control PC-3 spheres which were pretreated with cycloheximide (CHX) to inhibit the new protein synthesis. As shown in Fig. 5C, in CHX-pretreated spheres, there is no obvious difference in FZD6 protein level between luteolin-treated and control spheres (Fig. 5C). However, without CHX pretreatment, the protein level of FZD6 was upregulated by luteolin in the cells (Fig. 5C), which suggested that luteolin has no effect on the stability of FZD6. Taken together, these results demonstrated that luteolin upregulates the expression of FZD6 at the transcriptional level in PCa cells.

We next examined the effect of luteolin on Wnt signaling. As shown in Fig. 5D, luteolin significantly decreased the mRNA levels of the target genes (C-Myc and Cyclin D1) of Wnt signaling in both primary and PC-3 spheres, which indicated that luteolin inhibits Wnt signaling. To confirm this finding, TOP-flash plasmid (a luciferase reporter plasmid that contains two sets of 3 copies of the wild-type TCF binding regions) was transfected to primary PCa sphere-derived cells to monitor the effect of luteolin on the transcriptional activity of β-catenin (the transcriptional co-activator and the output of Wnt signaling) and the cells transfected with FOP-flash plasmid which contains mutated TCF binding regions were used as negative control. As shown in Fig. 5E, the results showed that luteolin significantly decreased the transcriptional activity of β-catenin, which confirmed that luteolin suppresses Wnt signaling in PCa cells.

To investigate the role of upregulation of FZD6 in luteolin-induced suppression of Wnt signaling, we generated FZD6-knockdown PC-3 stable cells (Supplementary Fig. S3A). As shown in Fig. 5F,G, the luteolin-induced decreased mRNA and protein levels of C-Myc and Cyclin-D1 were restored by FZD6 depletion in luteolin-treated PC-3 spheres. In addition, the decreased transcriptional activity of β-catenin was also recovered by knockdown of FZD6 (Supplementary Fig. S3B), which indicated that upregulation of FZD6 is the major mechanism underlying luteolin-induced suppression of Wnt signaling.

We subsequently examined the role of upregulation of FZD6 in the inhibitory effect of luteolin on PCa stemness in FZD6-knockdown PC-3 spheres. As shown in Fig. 5H,I, depletion of FZD6 abolished the inhibitory effect of luteolin on the spherogenicity and self-renewal capacity on serial passage in the cells. Moreover, the mRNA the protein levels of CD44 and CD133 were recovered by FZD6 silence in luteolin-treated spheres (Fig. 5J,K). These results demonstrated that upregulation of FZD6 is critical for luteolin suppressing the stemness of PCa cells.

As several studies indicated that GSK-3β is one of the targets of luteolin in several types of cancers^30,31^, we next investigated whether FZD6-mediated inhibitory effect of luteolin is GSK-3β-independent in prostate cancer. GSK-3β-knockdown PC-3 stable cell lines were developed (Supplementary Fig. S3C). As shown in Fig. 5J, luteolin significantly reduced the mRNA levels of C-Myc and Cyclin D1 in GSK-3β-depleted PC-3 cells. In addition, the transcriptional activity of β-catenin was also decreased by luteolin treatment in GSK-3β-depleted cells (Supplementary Fig. S3D), which demonstrated that luteolin inhibits Wnt signaling independently of GSK-3β in PCa cells.

Collectively, above results demonstrated that FZD6-mediated suppression of Wnt signaling is critical for luteolin inhibiting PCa stemness.

### FZD6 inhibits the stemness in prostate cancer

**Figure 6:**
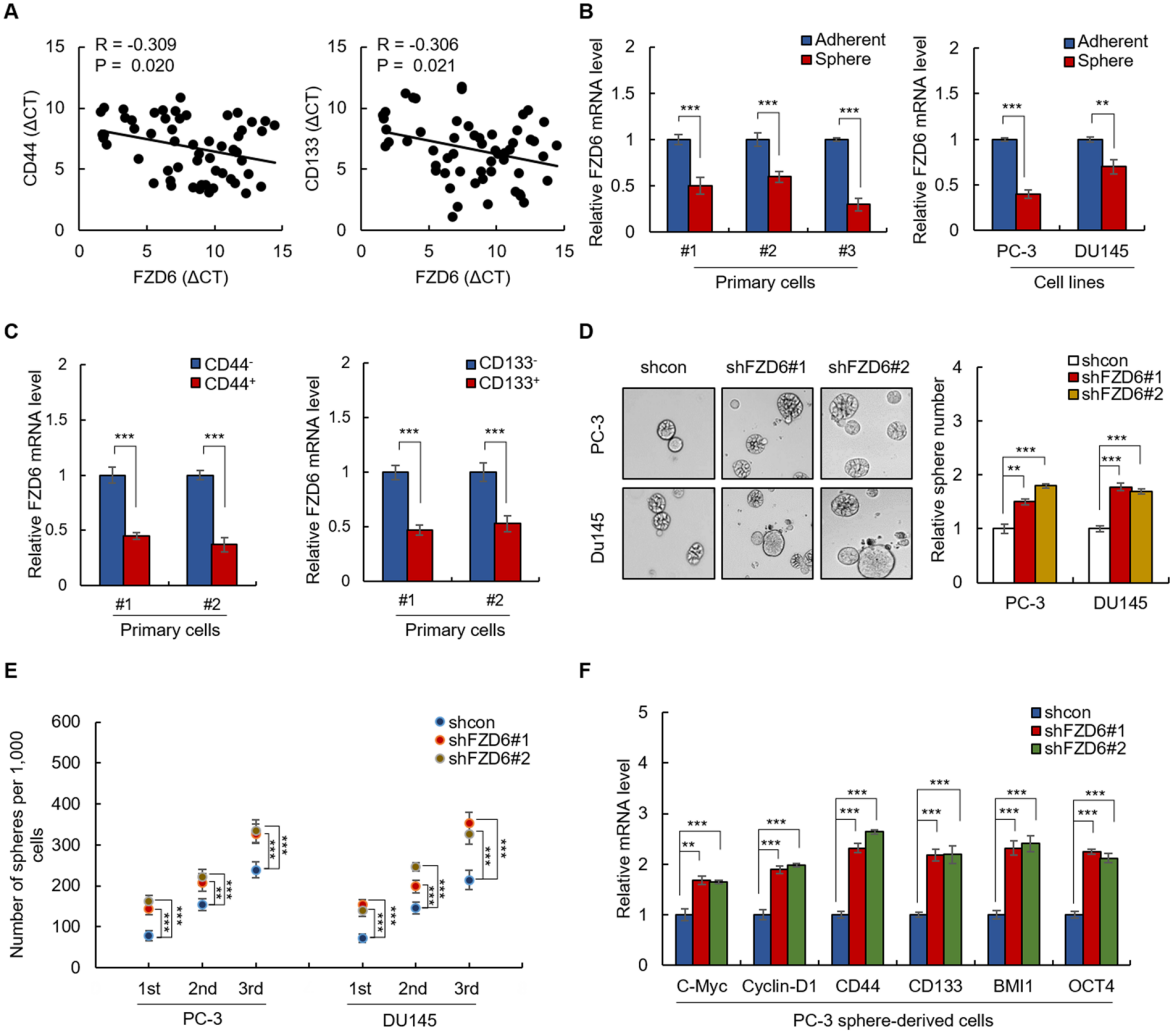
FZD6 suppresses the stemness of prostate cancer cells. (**A**) FZD6 is negatively correlated with CD44 and CD133 in spheres isolated from primary cultured prostate cancer cells. (**B**) qRT-PCR of FZD6 in spheres and adherent cells from primary PCa cells (left) and PCa cell lines (right). (**C**) The mRNA level of FZD6 in indicated primary PCa cells was analyzed by qRT-PCR. (**D**,**E**) FZD6 inhibits sphere formation activity of PCa cells. (**D**) Sphere formation assay of FZD6-depleted PC-3 cells and control cells was performed. (**E**) The number of 1st, 2nd and 3rd passaged spheres was counted. (**F**) qRT-PCR of indicated genes in FZD6-depleted PC-3 sphere and control cells. Data are representative of at least three independent experiments.

As the role of FZD6 in pathogenesis of prostate cancer has not yet been characterized and to further confirm our finding, we next examined whether FZD6 is a tumor suppressor in PCa and suppresses PCa stemness. We first examined the expression of FZD6 in a total of 43 prostate cancer tissues by qRT-PCR. As shown in Supplementary Fig. S4A, the expression of FZD6 is significantly downregulated in prostate cancer tissues compared with adjacent normal tissues. In addition, downregulation of FZD6 in tumor tissue predicts poor prognosis (Supplementary Fig. S4B). Furthermore, the expression of FZD6 is negatively correlates with PCa stem cell markers (CD44 and CD133) in spheres isolated from primary PCa cells (Fig. 6A). These results demonstrated that FZD6 serves as a tumor suppressor in PCa.

Next, we examined the effect of FZD6 on PCa stemness. As shown in Fig. 6B, the mRNA level of FZD6 was downregulated in primary spheres, compared to primary adherent cells. In addition, decreased mRNA level of FZD6 was observed in CD44^+^ and CD133^+^ primary PCa cells (Fig. 6C). These results indicated that FZD6 is downregulated in PCa stem cells. Next, we generated FZD6-knockdown PC-3 stable cells (Supplementary Fig. S4C) to confirm the inhibitory role of FZD6 in maintenance of the stemness of PCa cells. As shown in Fig. 6D,E, knockdown of FZD6 promoted the spherogenicity and self-renewal capacity of the cells. Furthermore, the mRNA levels of C-Myc, Cyclin D1, CD44 and CD133 were upregulated by FZD6 depletion (Fig. 6F), which indicated that FZD6 negatively regulates Wnt signaling and inhibits the stemness of prostate cancer.

Collectively, above results demonstrated that luteolin attenuates Wnt signaling via upregulation of FZD6 to suppress prostate cancer stemness.

## Discussion

In this study, by characterizing the changes in proteomic profiles of PC-3 cells with and without luteolin treatment, we identified novel potential targets and mechanisms of luteolin. We subsequently identified that FZD6 as a tumor suppressor suppresses the stemness of PCa cells. Furthermore, our results demonstrated that FZD6-mediated inhibition of Wnt signaling is critical for luteolin suppressing the prostate cancer stemness.

Recurrence and side effects of chemotherapy drugs are the bottleneck of prostate cancer therapy^2,3,9^. Anti-cancer drugs with reduced side effect are thus required for long-term therapy and prevention of prostate cancer^32^. Nature products have emerged as a promising source for novel anti-cancer drug development because of their relatively low toxicity and potential anti-cancer activity^10^. Among them, luteolin, a common dietary flavonoid, has been studied extensively as a potential anti-cancer agent^11,17,18^.

Luteolin inhibits initiation and progression of cancer cells by multiple mechanisms. For example, luteolin directly induces apoptosis by activating JNK in small cell lung cancer (SCLC) cells^33^. And, luteolin induces autophagy by accumulating microtubule-associated protein light chain-3 II protein in NCI-H460 lung carcinoma cells^34^. In MET4 metastatic squamous cell carcinoma (SCC) cells, luteolin triggers acidic lysosomal vacuolization to induces autophagy^35^. Furthermore, luteolin induces cell cycle arrest and the identified targets of luteolin in cell cycle pathway include, but not limited to, IGF1, PI3K/AKT, ERK, MAPK as well as FLT3 signaling pathways^36–38^.

In prostate cancer, some of the identified anti-cancer effects of luteolin were also observed. For example, we found that luteolin inhibits the phosphorylation of YB-1 in PC-3 cells (Supplementary Fig. S4D), which was reported in leukemic cells recently^13^. The regulation of essential cancer-related pathways, such as Wnt, Hippo, HIF-1, TNF and PI3K/AKT, etc., was also found in our proteomics results (Fig. 4). However, the exact mechanisms underlying these regulation in PCa could be different from that in other types of cancer. For example, in colorectal cancer, luteolin was reported to inhibit the translocation of β-catenin from the cytosol to the nucleus by modulating the expression of GSK-3β^31^, while, our results demonstrated that luteolin-induced inhibition of Wnt signaling in PCa cells is GSK-3β independent (Fig. 5J). In addition, suppression of transcription of β-catenin or its target gene, Cyclin D1, is supposed to be the major mechanisms underlying luteolin-induced inhibition of Wnt signaling in colorectal cancer^30^. However, in our study, we demonstrated that luteolin-induced upregulation of FZD6 is critical for luteolin suppressing Wnt signaling in PCa cells (Fig. 5). Thus, the exact mechanisms of luteolin targeting these pathways in specific type of cancer still needs to be further studied.

Wnt signaling pathway plays a fundamental role in cancer development. Aberrant activation of Wnt signaling pathway triggers upregulation of proto-oncogenes such as C-Myc and Cyclin D1^24,27^. Recent evidences indicated that Wnt signaling pathway is critical for maintenance of cancer stemness. Therefore, therapeutic targeting Wnt signaling pathway provides a strategy to suppress tumor growth. Through proteomics study, we found that FZD6 in Wnt signaling pathway was significantly upregulated by luteolin in PC-3 cells. And, FZD6 was identified as negative regulator of Wnt signaling by several studies. We thus hypothesized that upregulation of FZD6 is the mechanism underlying luteolin-induced suppression of Wnt signaling and the stemness in PCa cells. Indeed, we found that the inhibitory effect of luteolin on Wnt signaling pathway and the stemness of the cells was abolished by depletion of FZD6 (Fig. 5), which confirmed the hypothesis. As evidences showed that cancer stem cells contribute to chemoresistance of PCa^4–6^, we also examined the effect of luteolin on the sensitivity of PC-3 cells to docetaxel. As shown in Supplementary Fig. 4E, luteolin significantly sensitized PC-3 cell to docetaxel and this effect was abolished by FZD6 depletion. This result demonstrated the critical role of upreuglation of FZD6 in luteolin-induced chemosensitization and suggested the potential clinical application of luteolin as PCa chemosensitizer.

The role of FZD6 in cancer development is controversial. Colan and collaborators first reported that FZD6 is a negative regulator of canonical Wnt pathway in HEK293 cells^28^, which indicates that FZD6 may be a tumor suppressor. Consistent with this hypothesis, Huang *et al*. pointed that FZD6 attenuates Wnt pathway activity in glioblastoma^39^ and Yan *et al*. reported that FZD6, regulated by miR-21, represses gastric cancer cell proliferation and migration^40^. On the other hand, results from several studies indicated that FZD6 serves as oncogene that promotes cancer development. Corda *et al*. reported a genomic amplification of FZD6 in breast cancer^41,42^ and Cantilena *et al*. pointed that FZD6 could mark highly tumourigenic stem-like cells in mouse and human neuroblastomas^43^. These results promoted us to study the role of FZD6 in PCa. We subsequently found that the expression of FZD6 is suppressed in tumor tissues of PCa patients and down-regulation of FZD6 predicts poor prognosis (Supplementary Fig. S1A and S1B). In addition, the expression of FZD6 is negatively correlated with PCa stem cell markers and is suppressed in PCa spheres as well as CD44^+^ and CD133^+^ primary PCa cells (Fig. 6A–C). Moreover, FZD6 depletion promotes the spherogenicity and self-renewal capacity of PCa cells (Fig. 6D,E). Furthermore, depletion of FZD6 activates Wnt signaling and upregulates the expression of PCa stem cell markers (Fig. 6F). These results demonstrated that FZD6 as a tumor suppressor that can abolish PCa stemness and therefore confirmed that upregulation of FZD6 is the mechanism underlying luteolin-induced inhibition of Wnt signaling and the stemness in PCa cells.

Despite many studies, little was known about the regulatory effect of luteolin on the progress of gene transcription and translation. Microarray study from Kevin Shoulars *et al*. showed that luteolin regulates RNA transcription genes in PC-3 prostate cancer cells^44^. In this study, we also found that luteolin inhibits the expression of proteins involved in chromatin organization (Supplementary Table S1) and RNA processing (Supplementary Table S2). In addition, we also found that luteolin inhibits the expression of proteins involved in translation initiation, elongation, and termination (Supplementary Table S3). It suggests that luteolin may regulate gene expression level mainly by reducing synthesis but not by promoting degradation. This hypothesis was subsequently confirmed by measuring the half-life of FZD6 in luteolin-treated PCa cells which pretreated with CHX to inhibit new protein synthesis (Fig. 5C). This finding largely improved our understanding of mechanism-of-action of luteolin that the regulated expression of many previously recognized luteolin target genes were the secondary event that is due to the inhibition of transcription and translation process.

In summary, our results provided the evidence of novel mechanisms of luteolin against prostate cancer cell including regulation of Wnt/β-catenin pathway. We also revealed that FZD6, one of the targets of luteolin, plays a tumor suppressive role in prostate cancer. Our findings shed light on the mechanisms and pathways through which luteolin may exert its anti-prostate cancer effects during cancer initiation and progression, which would be important for translational application of luteolin and for the design of more effective drugs. Our work also suggests a new therapeutic strategy against prostate cancer caused by aberrant activation of Wnt signaling.

## Methods

### Cell culture and treatment

Human prostate cancer cell lines (PC-3, DU145) were purchased from the American Type Culture Collection (ATCC) and were grown in RPMI 1640 (GE Healthcare Life science) with 10% heat-inactivated fetal bovine serum (Life Technologies), 100 units/mL penicillin, and 100 μg/mL streptomycin, in 5% CO2 incubator at 37 °C. Lenti-X 293 T cell line was purchased from Clontech Laboratories Inc. Luteolin was dissolved in dimethyl sulfoxide (DMSO) at 10 mM (stock solution) and was stored in several aliquots at −20 °C to avoid repetitive freeze-thaw cycles. To prepare working solutions, the luteolin stock solution was serially diluted using DMSO. Working solutions were diluted 100 times with culture media before treatment. For proteomic analysis, 1 × 10^7^ cells were seeded into 100 mm dishes and allowed to attach for 24 h. At this time, the media was changed and the cells were grown for 24 hours in growth media containing 5 μl of DMSO (controls) or luteolin in 5 μl of DMSO (5 μM final concentration). After incubation, 1 × 10^7^ cells for each treatment group were harvested for protein extraction.

### Clinical samples

All patient samples were obtained from Oncology Department, East Campus of Shanghai Jiao Tong University Affiliated Sixth People’s Hospital with written informed consent. The ethical approval was granted from Committees for Ethical Review in Shanghai Jiao Tong University and all experiments in this study were performed in accordance with the relevant guidelines.

### Primary prostate cancer sphere-derived cells culture

Prostate cancer tissues from different areas of the tumor were dissected and washed with Hank’s balanced salt solution for several times to remove the blood and contaminant. The fat and necrotic tissues were then removed by sterile forceps. The prostate cancer tissues were minced into pieces of 1 mm^3^ and maintained in serum-free medium supplemented with 2% B-27 supplement (Invitrogen), 20 ng ml^−1^ FGF2 and 20 ng ml^−1^ EGF. The collagenase (40 U ml^−1^) was added to medium for digestion and the dissociated tissues were then passed through 100 μM cell strainer filter. Erythrocytes were removed by treatment with DB Pharm lyse lysing buffer (BD Falcon) for 1 min. The resulting cells were washed by centrifuge for several times and placed into Ultra-Low attachment 6-well plates (Corning) for sphere formation.

### Plasmid, primers, shRNAs and antibodies

For knockdown of FZD6 and GSK-3β, shRNAs against FZD6 and GSK-3β were cloned into PLKO.1 vector and the recombinant constructions were identified by Sanger DNA sequencing. TOP-FLASH luciferase reporter plasmid that contains two sets of 3 copies of the wild-type TCF binding regions was used for monitoring the transcriptional activity of β-catenin and FOP-FLASH with mutated TCF binding region was used as negative control. Primers, shRNAs and antibodies used in this study were listed in Supplementary Tables S4, S5 and S6. Luteolin, Polyethylenimine (PEI), Acetonitrile (ACN, HPLC-grade), formic acid (HPLC-grade), water (HPLC-grade), sodium dodecyl sulfate (SDS), Tris (2-carboxyethyl) phosphine hydrochloride (TCEP) and Iodoacetamide (IAA) were purchased from Sigma-Aldrich. Bio-Rad DC protein assay reagents and bovine serum albumin standard were obtained from Bio-Rad Laboratories. iTRAQ reagents kit and trypsin were purchased from AB SCIEX. 30 kDa cut-off filter membrane was from Pall Corporation. Tri-HCl and ammonium bicarbonate were from FASP kit. UltraMicroSpin column was bought from The Nest Group. Other reagents were purchased from Sigma-Aldrich.

### Cell proliferation assay

The proliferation of prostate cancer cells was examined using CCK-8 kit (Dojindo Laboratories, Kumamoto, Japan) according to the manufacturer’s instructions. Briefly, the cells were seeded into 96-well cell culture plates at a density of 5 × 10^3^ cells/well in 200 µl cell culture medium containing DMSO control or luteolin and cultured for 48 h. Then CCK-8 solution was added into each well and incubated for 4 h. The absorbance (A) at 450 nm was measured using a plate reader. The results from three individual experiments, each performed in triplicate were summarized.

### Drug sensitivity assay

The cells were seeded at a density of 5,000 cells/well in 96-well plate. Twenty-four hours after seeding, the cells were exposed to serially diluted luteolin (Fig. 5A) and incubated for another 24 h. The cell number was determined as mentioned above. Inhibition rate was calculated according to following formula: Inhibition % = 1 - (A_treatment_-A_blank_)/(A_vehicle_-A_blank_) %. IC_50_ values were calculated using non-linear regression analysis. Three independent experiments, each done in triplicate, were performed.

### Cell migration assay (Transwell Cell Migration Assay)

Complete culture media was added into the lower wells of a CytoSelect Fluorometric 8 µM Transwell Migration Assay plate (Cell Biolabs San Diego, CA, USA). Then cells were suspended in basal media and added into the upper wells of the plate with vehicle (DMSO) or luteolin followed by incubation for another 24 h to allow cells to migrate across the porous membrane. Cells in the upper wells were discarded. Migrated cells on the lower face of transwell membrane were detached and lysed. Total DNA in the resulting lysates was then stained by CyQuant GR dye (Millipore, Billerica, MA) and quantified by fluorometric analysis according to manufacturer’s instructions. The results from three individual experiments, each performed in triplicate, were summarized.

### Cell migration assay (Wound healing assay)

Cell migration was also analyzed in a wound healing assay using IBIDI culture inserts (IBIDI GmbH, Martinsried, Germany) according to the manufacturer’s manual. In brief, an IBIDI culture insert was placed into one well of the 24 well plate and slightly pressed to ensure tight adhesion. Then, 1.0 × 10^5^ cells in 70 μl complete culture media were added into the inserts. After 24 h, the insert was removed, creating a gap of 500 μm. The migration was documented under a light microscope.

### Colony formation assay

Cells were seeded into 6-well cell culture plates and allowed to attach overnight. Then the media was replaced with complete media containing vesicle (DMSO) or luteolin. After 15–20 days of culture, colonies were stained with crystal violet and colonies with >50 cells were counted. Three independent experiments, each done in triplicate, were performed.

### Sphere formation assay

The cells were seeded into Ultra-Low attachment 6-well plates (Corning). After 10–15 days culture, the spheroids were observed under a phase-contract microscopy (Leica).

### Sample preparation for iTRAQ quantitative proteomic analysis

Cell pellet containing 1 × 10^7^ cells was re-suspended in 1 ml of lysis solution (acetonitrile:50 mM ammonium bicarbonate at the ratio of 1:9, v/v), and 1 mg of ProteaseMax powder (V2072, Promega, WI, USA) was added into 1 ml of lysis solution to make up a final concentration of 0.1% w/v. The cell lysate was centrifuged at 14,000 rpm for 7 min at room temperature and the supernatant was used for measuring total protein concentration using Bio-Rad DC protein assay (Bio-Rad, California, USA). An aliquot of 100 μg total protein from each sample was used for iTRAQ 4-plex experiment. The experimental design for the iTRAQ 4-plex is given in Fig. 2A.

Next, 50 mM of TCEP was added to the denatured proteins and incubated for 1 h at 60 °C. Samples were then cooled to room temperature before transferring to 30 kDa cut-off filter membrane; 75% urea solution was added to the membrane and centrifuged to remove SDS. 15 mM of IAA (final concentration) was added to alkylate the reduced proteins for 30 minutes at room temperature in the dark. The samples were washed three times with 75% urea solution followed by 0.1 M Triethylammonium bicarbonate solution (TEAB). Trypsin was added in a ratio of 1:50 and incubated for 16 h at 37 °C. The peptides were eluted with 0.1 M TEAB and 0.5 M sodium chloride and dried prior to addition of iTRAQ labeling reagents. The iTRAQ reagent was added into the respective samples and incubated at room temperature for 2 h. The labeled samples were pooled together for subsequent high pH reverse phase fractionation.

### High pH reverse phase (RP) fractionation

The 4-plex combined samples were reconstituted in mobile phase A (20 mM ammonium formate, pH 10). The sample was injected into Waters HPLC system (Elstree, UK) using PDA photodiode array detector (Elstree, UK). Waters Acquity UPLC BEH C18, 3.5 μm, 3.0 × 150 mm column was used. A step linear gradient of mobile phase B (80% Acetonitrile in 20 mM Ammonium formate, pH 10) from 5–15% over 20 min, 15–40% over 20 min, 40–80% over 1 min at a flow rate of 0.4 mL/min was utilized for this fractionation step. A total of 10 fractions were collected for subsequent nano-LC-MS/MS analysis.

### NanoLC-MS/MS analysis

Peptide mixture from each fraction (total 10 fractions) was reconstituted in 12 μl of loading buffer (mobile phase A: 0.1% formic acid, 2% acetonitrile in water), and 2 μl from each fraction was injected into Ultimate 3000 nanoLC system (Dionex, Thermo Fisher Scientific, MA, USA) coupled with AB Sciex 5600 TripleTOF (AB Sciex, Framingham, MA, USA) for the analysis. Each fraction was injected twice (technical replicates). A 15 cm × 75 µm i.d. packed with Acclaim PepMap RSLC C18 column (Dionex, Thermo Fisher Scientific, MA, USA) was used for peptide separation. A spray tip (New Objectives, Woburn, MA) was used to connect the nano-column with the nano-spray interface into AB Sciex 5600 TripleTOF mass spectrometer. Samples were loaded onto a trap column (Acclaim PepMap 100 C18, 2 cm × 75 µm i.d., Dionex, Thermo Fisher Scientific, MA, USA) at a flow rate of 5 µL/min. After a 5 min wash with loading buffer (2/98 v/v of ACN/water with 0.1% formic acid), the system was switched into line with the C18 analytical capillary column. For the analysis, a step linear gradient was used according to the following program: mobile phase B (2/98 v/v of water/ACN with 0.1% formic acid) from 5–12% over 30 min, 12–40% over 60 min, and 40–90% over 20 min at a flow rate of 300 nL/min.

The settings for the mass spectrometer were as follows: Ionspray Voltage Floating (ISVF) = 2000 V, curtain gas (CUR) = 30, Ion source gas 1 (GS2) = 10, Interface Heater Temperature (IHT) = 125, declustering potential (DP) = 100 V, Nebuliser current (NC) = 3 for nitrogen gas. Information-dependent acquisition (IDA) mode with Analyst TF 1.7 software (AB Sciex, USA) was used for data acquisition. For IDA parameters, 0.25 s time-of-flight mass spectrometry survey scan in the mass range of 400–1250 was followed by product ion scan of 0.05 s in the mass range of 100–1500. Switching criteria were set as follows: mass range: 400 to 1250; charge state: 2~5; maximum number of candidate ions to monitor per cycle: 40 spectra; abundance threshold: >120 counts. Former target ions were excluded for 12 s. IDA Advanced settings included “rolling collision energy (CE)”, “adjust CE when using iTRAQ Reagent”, and “dynamic accumulation”.

### Proteomics data analysis and statistical analysis

ProteinPilot 5.0 software (AB SCIEX) was used for protein identification and iTRAQ-based relative quantification. The protein database (version: uniprot_all_Oct2014) was used for data searching. The typical settings in ProteinPilot were as follows: (a) sample type: iTRAQ 4 plex (peptide labeled); (b) cys alkylation: iodoacetamide; (c) digestion: trypsin; (d) instrument: TripleTOF 5600; (e) special factors: none; (f) ID focus: biological modifications; (g) search effort: thorough ID; and (h) bias correction and background correction. 95% confidence level was used at the peptide level. FDR <1% in the ProteinPilot software was used for protein identification. Reverse database search strategy was used to calculate FDR for peptide identification. iTRAQ based relative quantitation was based on peak areas of reporter ions using Pro Group algorithm in ProteinPilot. Auto bias correction was applied to eliminate possible pipetting errors in the combination of labeled samples. The strategy for further data analysis of considering technical and biological replicates is given in Fig. 2B.

### Bioinformatics analysis

Pathway analysis was performed using iPathwayGuide^25^ online bioinformatics tool (https://apps.advaitabio.com)^26^. Gene ontology classification was performed by both iPathwayGuide and David 6.7 online software (https://david.ncifcrf.gov/home.jsp).

### Immunoblotting

The cells were lysed in RIPA buffer (Thermo). Lysates were electrophoresed on SDS-PAGE (10% polyacrylamide gels) and transferred to polyvinylidene difluoride membrane (Millipore) by semidry blotting. Membranes were blocked for 1 hour at room temperature with 5% nonfat dry milk in TBS containing 0.05% Tween-20. The membranes were then incubated overnight at 4 °C in blocking buffer with the indicated antibodies. Membranes were washed in blocking buffer and incubated with secondary antibody conjugated to horseradish peroxidase. Visualization of the protein bands was done using Clarity western ECL substrate chemiluminescent detection reagent (Bio-Rad) according to manufacturer’s instruction.

### Quantitative real-time reverse-transcription PCR

Quantitative real-time reverse transcription PCR analysis was performed using total RNA and the SYBR green reagent (Takara) with an ABI stepOne real-time pcr system. The results were determined using the comparative Ct method with the housekeeping gene GAPDH as a control.

### Lentiviral generation and infection

Lentiviruses were created by co-transfecting HEK293T cells with expression plasmid, a packing plasmid (delta R8) and envelop plasmid (VSV-G). Media with lentivirus particles were used to infect the PC-3 and DU-145 cells. The stable cell line was selected by puromycin for 10 days. Knockdown efficiency was determined by Western blot analysis.

### Luciferase reporter assay

The cells were co-transfected with reconstructed luciferase reporter vector and hRluc/TK vector. Luciferase activity was detected with Dual-Luciferases Reporter Assay kit (Promega). Relative firefly luciferase activity was normalized to renilla luciferase activity.

### Statistical analysis

The data are represented as the mean ± SD from three independent experiments except where indicated. Data analysis was performed using the Student’s *t*-test (unpaired, two-tailed) on raw data with SPSS at a significance level of P < 0.05. *P < 0.05, **P < 0.01, ***P < 0.001.

## Electronic supplementary material

Supplementary information
