# Supplementary material for: Luteolin attenuates Wnt signaling via upregulation of FZD6 to suppress prostate cancer stemness revealed by comparative proteomics
Source: Sci Rep. 2018 Jun 4;8:8537. doi: 10.1038/s41598-018-26761-2 (PMC5986741; doi:10.1038/s41598-018-26761-2)
Supplement: Supplementary file 1 — Supplementary information [file 41598_2018_26761_MOESM1_ESM.pdf]

Supplementary information

Luteolin attenuates Wnt signaling via upregulation of FZD6 to suppress prostate cancer stemness revealed by comparative proteomics

Kun Han[1*], Tingyuan Lang[2*], Zhiqi Zhang[1], Yi Zhang[2], Yongning Sun[1], Zan Shen[1], Roger W. Beuerman[3,4,5], Lei Zhou[3,4,5] & Daliu Min[1]

[1]Department of Medical Oncology, The Affiliated 6th People's Hospital of Shanghai Jiaotong University, Shanghai 200233, China. [2]Key Laboratory of Clinical Laboratory Diagnostics (Ministry of Education), College of Laboratory Medicine, Chongqing Medical University, Chongqing 400016, China. [3]Singapore Eye Research Institute, The Academia, 20 College Road, Discovery Tower level 6, Singapore 169856, Singapore. [4]Department of Ophthalmology, Yong Loo Lin School of Medicine, National University of Singapore, 1E Kent Ridge Road, NUHS Tower Block Level 7, Singapore 119228, Singapore. [5]Neuroscience and Behavioral Disorders Program, Duke-NUS Graduate Medical School, 8 college Road, Singapore 169857, Singapore.

*These authors contributed equally to this work. Correspondence and requests for materials should be addressed to Daliu Min (email: doctor_mindaliu@163.com) or Lei Zhou (email: zhou.lei@seri.com.sg)

Supplementary Figures

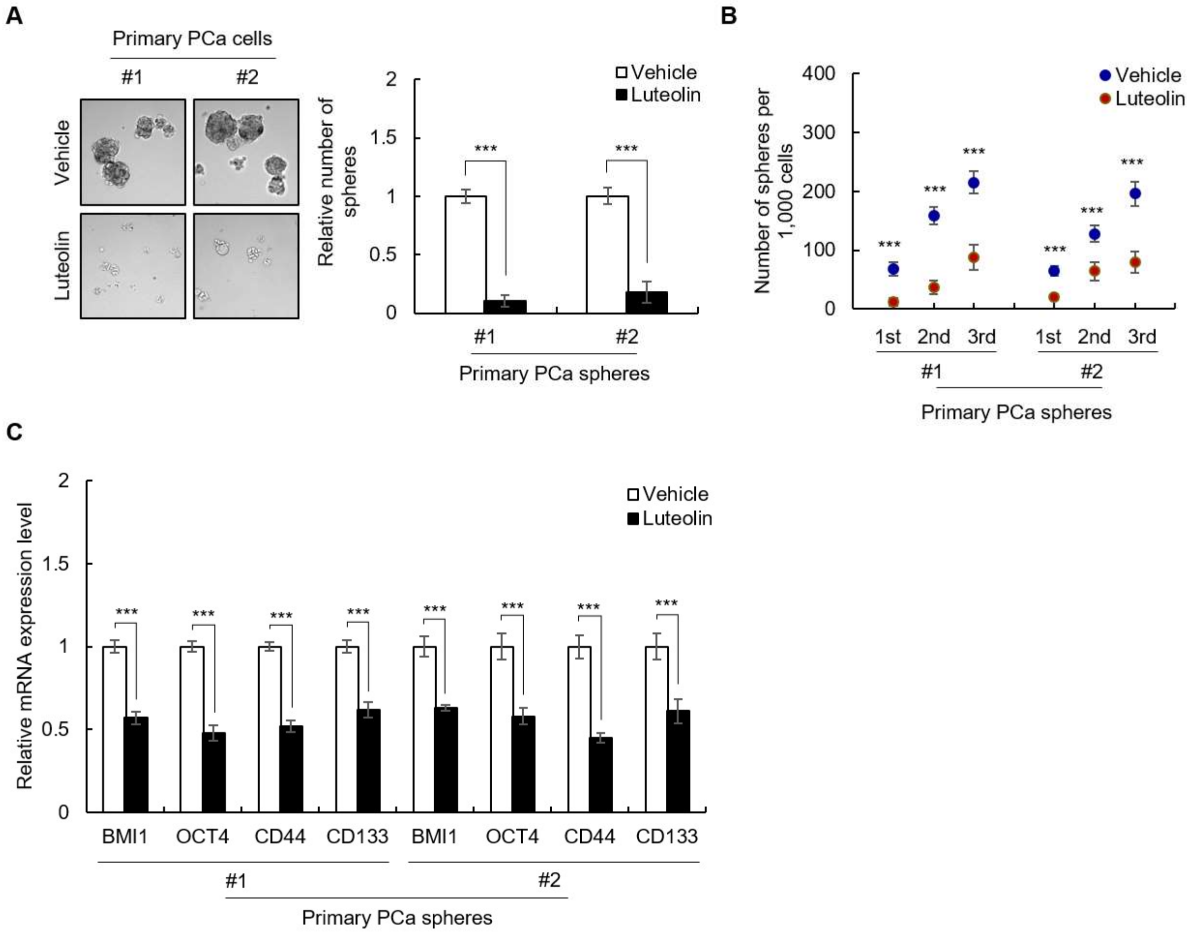

Figure S1. Luteolin inhibits the stemness of PCa primary cells. (A) Luteolin inhibits sphere formation of primary PCa cells. Primary PCa cells were treated with 5μM of luteolin or equal volume of vehicle for 15 days. Sphere formation assay was performed. **(B)** Luteolin inhibits self-renewal of primary PCa cells. Sphere formation assay was performed on lutoelin-treated or non-treated primary PCa sphere-derived cells at passage 1 through 3 for 15 days. **(C)** Luteolin inhibits the expression of cancer stem cell markers in primary PCa spheres. Spheres were isolated from primary PCa cells and treated with or without luteolin for 24 h. Total RNA were prepared for qRT-PCR analysis. Data are representative of at least three independent experiments.

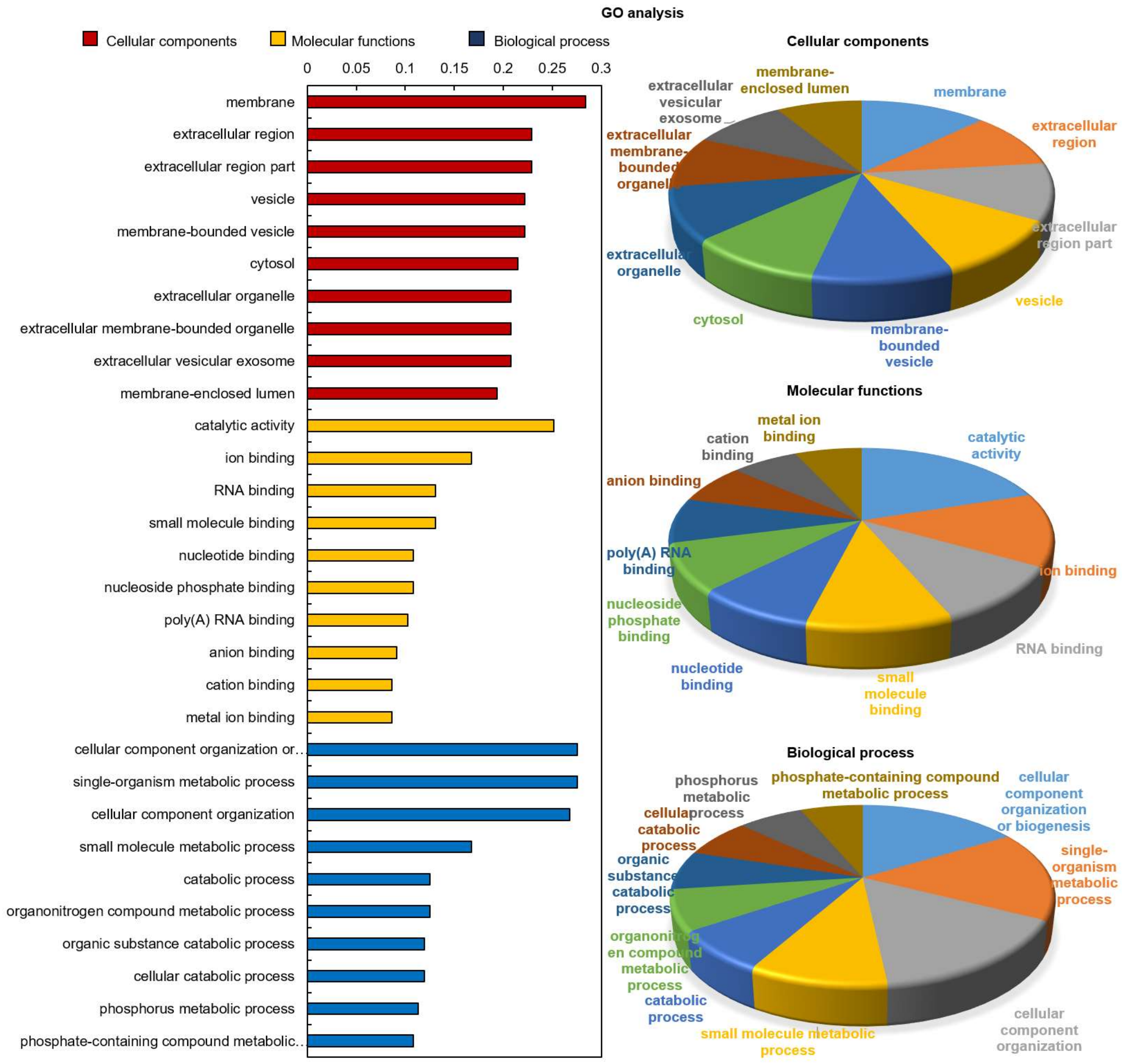

Figure S2. Gene Ontology analysis of the differentially expressed genes. The gene symbols of 208 differentially expressed proteins were subjected to iPathwayGuide online software for Gene Ontology analysis. Top 10 impacted gene ontology terms in every category (Cellular components, Molecular functions, Biological process) were listed.

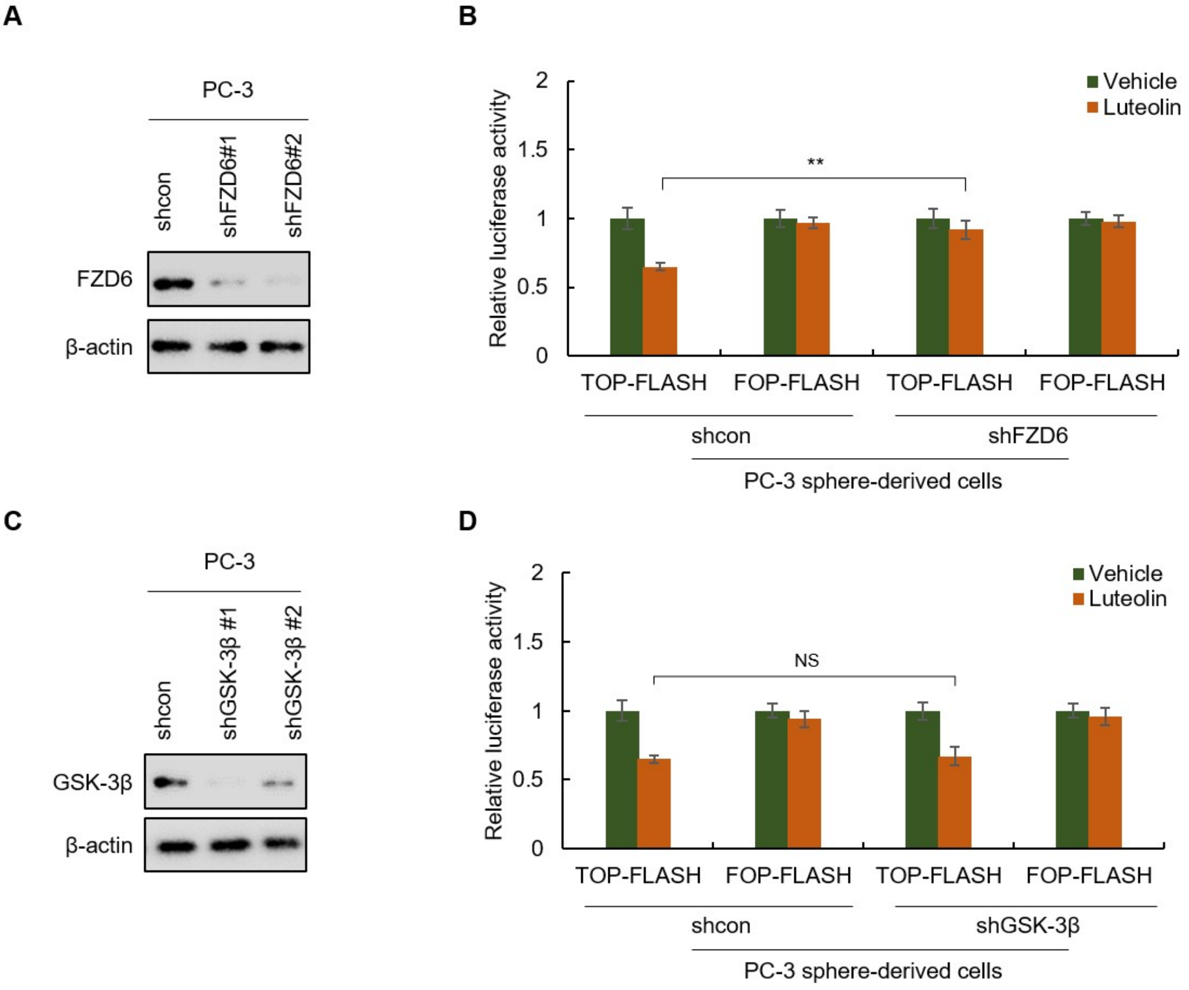

Figure S3. FZD6-mediated suppression of Wnt signaling is critical for luteolin inhibiting PCa stemness (related to Figure 5). (A,C) Characterization of FZD6-depleted **(A)** and GSH-3β-depleted **(C)** PC-3 stable cell line. **(B,D)** Indicated cells were transfected with TOP-FLASH and FOP-FLASH plasmid, which were followed by luteolin or vehicle treatment. Luciferase activity was measured 24 h after treatment.

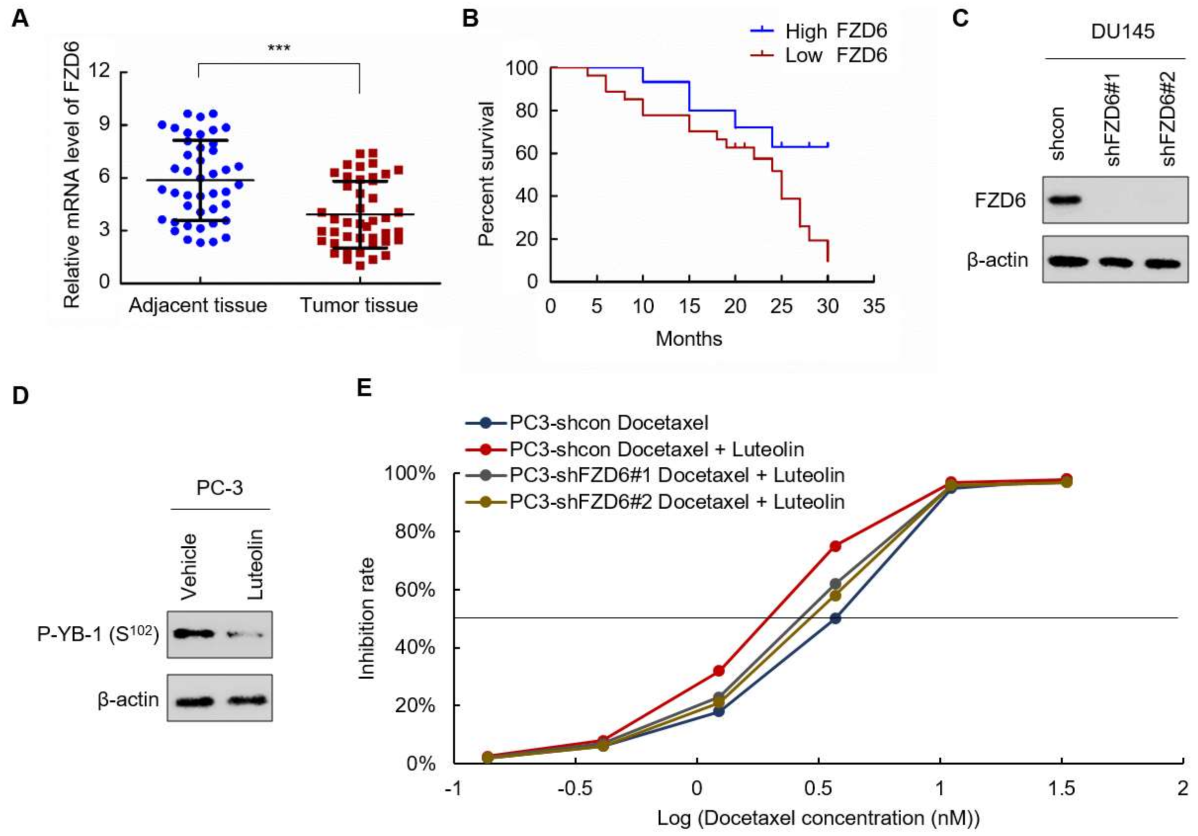

Figure S4. FZD6 suppresses stemness and Wnt signaling pathway in PCa (related to Figure 6). **(A)**The mRNA level of FZD6 in tumor tissue and adjacent normal tissue of prostate cancer patients was analyzed by quantitative real-time PCR. **(B)** Kaplan-Meier analysis of overall survival of prostate cancer patients in low and high FZD6 groups. **(C)** Characterization of FZD6-knockdown DU145 cells. **(D)** Luteolin reduced the phosphorylation level of YB-1 in PC-3 cells. Total protein in PC-3 cells treated with luteolin or equal volume of vehicle was prepared for western blot analysis. **(E)** Upregulation of FZD6 is necessary for luteolin sensitizing PC-3 cells to docetaxel. Limiting dilution analysis was performed to determine the sensitivity of FZD6-knockdown PC-3 cells and control cells to docetaxel alone and combination of docetaxel and luteolin.

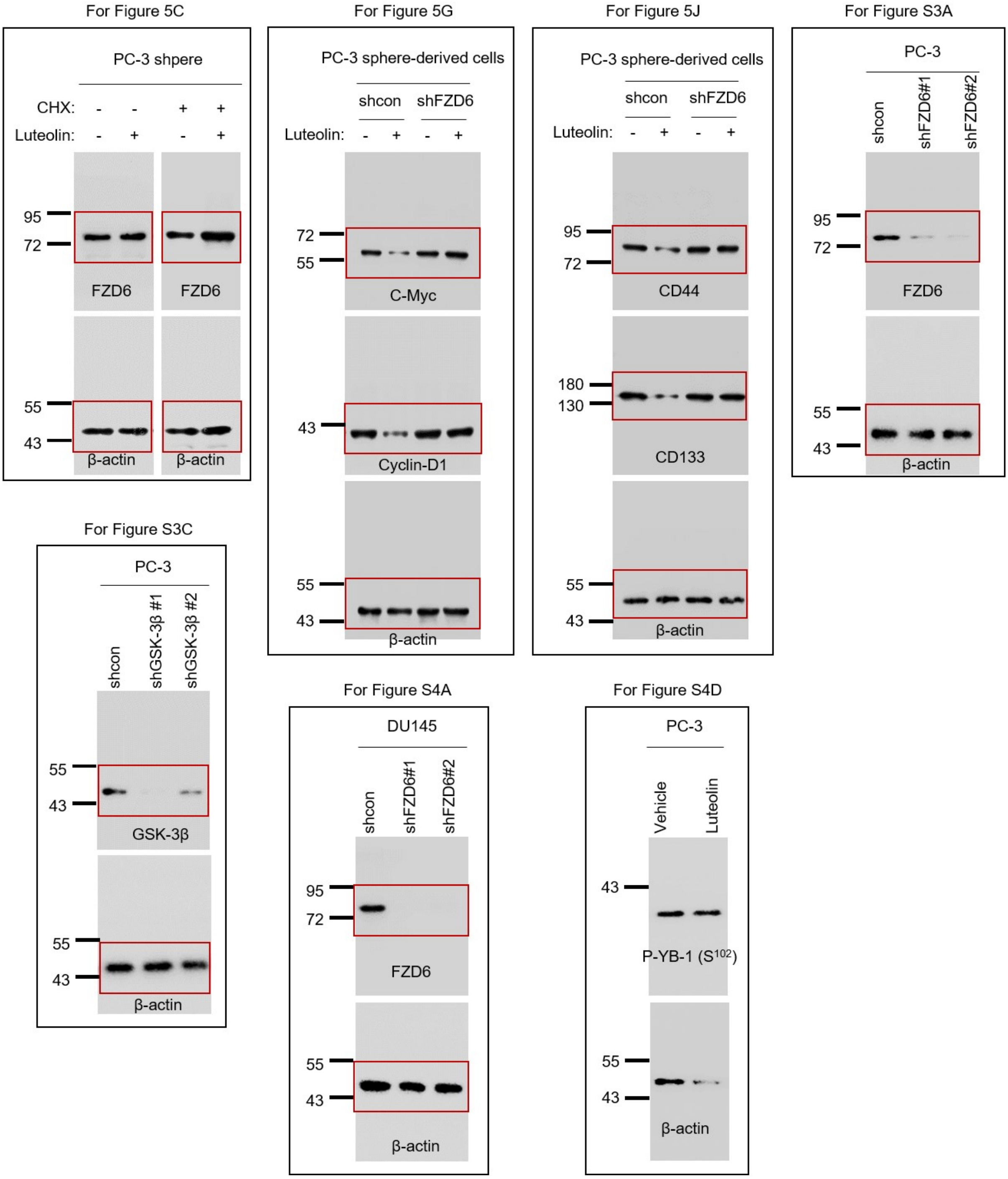

Figure S5. Uncropped versions of the blots in indicated Figures.

Supplementary Tables

Table S1: Identified 11 proteins involved in chromatin organization.

**Gene symbol**	**Protein name**	**Up/down-regulated by luteolin**
DNAJC2	DnaJ (Hsp40) homolog, subfamily C, member 2	up
PAXBP1	PAX3- and PAX7-binding protein 1	up
WDR61	WD repeat domain 61	down
BRD7	bromodomain containing 7; bromodomain containing 7 pseudogene 2	down
IPO4	importin 4	down
SKP1	S-phase kinase-associated protein 1	down
KDM4B	lysine (K)-specific demethylase 4B	down
CAMK2D	calcium/calmodulin-dependent protein kinase II delta	down
PHB	prohibitin	down
OTUB1	OTU domain, ubiquitin aldehyde binding 1	down
NAP1L1	nucleosome assembly protein 1-like 1	down

NOTE: Gene ontology term: Chromatin organization.

Table S2: Identified 13 proteins involved in mRNA processing.

Gene symbol	Protein name	Up/down-regulated by luteolin
YBX1	Y box binding protein 1	up
METTL3	methyltransferase like 3	up
DDX23	DEAD (Asp-Glu-Ala-Asp) box polypeptide 23	up
SYNCRIP	synaptotagmin binding, cytoplasmic RNA interacting protein	down
SF3A3	splicing factor 3a, subunit 3, 60kDa	down
PPWD1	peptidylprolyl isomerase domain and WD repeat containing 1	down
EFTUD2	elongation factor Tu GTP binding domain containing 2	down
TRA2B	transformer 2 beta homolog (Drosophila)	down
HNRNPF	heterogeneous nuclear ribonucleoprotein F	down
C1QBP	complement component 1, q subcomponent binding protein	down
PCBP1	poly(rC) binding protein 1	down
PTBP1	polypyrimidine tract binding protein 1	down
TUT1	terminal uridylyl transferase 1, U6 snRNA-specific	down

NOTE: Gene ontology term: mRNA processing.

Table S3: Identified nine proteins involved in translation initiation, elongation and termination.

Gene symbol	Protein name	Up/down-regulated by luteolin
RPL22	ribosomal protein L22	up
RPS28	ribosomal protein S28	up
FAU	Finkel-Biskis-Reilly murine sarcoma virus (FBR-MuSV) ubiquitously expressed	down
RPL7	ribosomal protein L7	down
RPL17	ribosomal protein L17	down
RPS7	ribosomal protein S7	down
EIF5A	eukaryotic translation initiation factor 5A	down
EEF1G	eukaryotic translation elongation factor 1 gamma	down
HSPB1	heat shock protein family B (small) member 1	down

NOTE: Gene ontology term: Translation initiation, Translation elongation, Translation termination.

Table S4. Primers used in this study

Quantitative real-time reverse-transcription PCR			
C-Myc	F: 5’-CGTCTCCACACATCAGCACAA-3’	***Cyclin-D1***	F: 5’-GGCGGAGGAGAACAAACAGA-3’
	R: 5’-CACTGTCCAACTTGACCCTCTT-3’		R: 5’-TGGCACAAGAGGCAACGA-3’
CD44	F: 5’-CTGCCGCTTTGCAGGTGTA-3’	***CD133***	F: 5’-AGTCGGAAACTGGCAGATAGC-3’
	F: 5’-CATTGTGGGCAAGGTGCTATT-3’		F: 5’-GGTAGTGTTGTACTGGGCCAAT-3’
BMI1	F: 5’-CGTGTATTGTTCGTTACCTGGA-3’	***OCT4***	F: 5’-CTGGGTTGATCCTCGGACCT-3’
	F: 5’-TTCAGTAGTGGTCTGGTCTTGT-3’		F: 5’-CCATCGGAGTTGCTCTCCA-3’
GAPDH	F: 5’-AATCCCATCACCATCTTCCA-3’		
	R: 5’-TGGACTCCACGACGTACTCA-3’		
Reverse-transcription PCR			
FZD6 promoter(-1000-+155)	F: 5’-GGTGCTGGGGAGGCAACGGCGGGAC-3’		
	R: 5’-CTCTGGTCATAATGACCCAT-3’		

Table S5. shRNAs used in this study

shFZD6#1	5’-TGCTGTTGACAGTGAGCGAGGCTTGTATCTTGTGCCATTATAGTGAAGCCACAGATGTATAATGGCACAAGATACAAGCCGTGCCTACTGCCTCGGA-3’
shFZD6#2	5’-TGCTGTTGACAGTGAGCGACCAGAGAGACCAATTATATATTAGTGAAGCCACAGATGTAATATATAATTGGTCTCTCTGGGTGCCTACTGCCTCGGA-3’
shGSK3β#1	5’-TGCTGTTGACAGTGAGCGCGGAGAACCCAATGTTTCGTATTAGTGAAGCCACAGATGTAATACGAAACATTGGGTTCTCCTTGCCTACTGCCTCGGA-3’
shGSK3β#2	5’-TGCTGTTGACAGTGAGCGCGCTGTTACTAGGACAACCAATTAGTGAAGCCACAGATGTAATTGGTTGTCCTAGTAACAGCTTGCCTACTGCCTCGGA-3’
shluc	5’-TGCTGTTGACAGTGAGCGCGCTGAGTACTTCGAAATGTCTAGTGAAGCCACAGATGTAGACATTTCGAAGTACTCAGCGTGCCTACTGCCTCGGA-3’

Table S6. Antibodies used in this study

Antigen	***Application***	***Supplier***	***Catalog #***	***Dilution***
**FZD6**	IB	Cell signaling technology	#5158	1000
**C-Myc**	IB	Cell signaling technology	#13987	1000
**Cyclin-D1**	IB	Cell signaling technology	#2978	1500
**CD44**	IB	Cell signaling technology	#3570	1000
**CD133**	IB	Cell signaling technology	#64326	1500
**GSK-3β**	IB	Cell signaling technology	#12456	1500
**P-YB-1S^{102}**	IB	Cell signaling technology	#2900	1500
**β-actin**	IB	Cell signaling technology	#4970	1000
